# A Novel Method for Non-invasive Estimation of Primary Productivity in Aquatic Ecosystems Using a Chlorophyll Fluorescence-Induced Dynamic Curve

**DOI:** 10.3389/fmicb.2021.682250

**Published:** 2021-06-14

**Authors:** Hui Chen, Kunfeng Li, Chunling Xue, Qiang Wang

**Affiliations:** ^1^Key Laboratory of Algal Biology, Institute of Hydrobiology, Chinese Academy of Sciences, Wuhan, China; ^2^State Key Laboratory of Crop Stress Adaptation and Improvement, School of Life Sciences, Henan University, Kaifeng, China; ^3^University of Chinese Academy of Sciences, Beijing, China

**Keywords:** algal blooms, aquatic ecosystems, cell density, chlorophyll *a* content, chlorophyll fluorescence induced dynamic curve, photosynthetic microalgae, water primary productivity

## Abstract

Photosynthetic microalgae are a major contributor to primary productivity in aquatic ecosystems, but typical measurements of their biomass and productivity are costly and relatively inefficient. The chlorophyll fluorescence induced dynamic (OJIP) curve can reflect the original photochemical reaction and the changes to the function and structure of photosystems as well as the effects of environmental factors on photosynthetic systems. Here, we present a novel method for estimating the Chl *a* content and photosynthetic microalgal cell density in water samples using the integral area of the OJIP curve. We identify strong linear relationships between OJIP curve integrals and both Chl *a* contents and cell densities for a variety of microalgal cultures and natural communities. Based on these findings, we present a non-invasive method to estimate primary productivity in aquatic ecosystems and monitor microalgal populations. We believe that this technique will allow for widespread, rapid, and inexpensive estimating of water primary productivity and monitoring of microalgal populations in natural water. This method is potentially useful in health assessment of natural water and as an early warning indicator for algal blooms.

## Introduction

Microalgae are the main component of plankton biomass in aquatic ecosystems and are widely distributed across various aquatic environments (Carrick and Schelske, 1997). Photosynthetic microalgae also contribute to primary productivity in open fresh and ocean waters (Iriarte and Purdie, 1994), where algal photosynthesis contributes to more than 50% of the total global primary productivity and forms the basis of almost all of the aquatic food webs (Schnitzer et al., 1993; Parlevliet and Moheimani, 2014). As a result, microalgal biomass is a useful proxy for primary productivity in many aquatic systems.

There have been numerous attempts to determine primary productivity in natural aquatic systems (Nielsen, 1952; Odum, 1956; Goldman, 1968; Vollenweider, 1968). Currently, common methods include the ^14^C method and the oxygen method (Lipkin et al., 1986; Yang et al., 2002). The C^14^ tracer method applied in assessment of photosynthetic algae photosynthesis is the earliest and most classic technique for determining water primary productivity (Nielsen and Jensen, 1957). In the ^14^C method, carbon assimilation from photosynthesis is estimated and related to pure primary productivity (Williams et al., 1983). Although the ^14^C method is relatively sensitive and flexible, its operation time is costly due to long incubation periods (Hall and Moll, 1975). In the oxygen method, O_2_ released in photosynthesis is related to primary productivity. However, the oxygen method has several shortcomings; for instance, it is challenging to estimate gas exchange coefficients, and this technique is both time-consuming and has low sensitivity (Bott et al., 1978). An alternative approach to assessing productivity involves estimating photosynthesis based on chlorophyll (Chl) *a* concentration. Chl *a* is a reliable indicator for microalgal biomass and reflects the water quality status. Based on Chl *a* content, primary productivity in natural aquatic systems can be calculated when combined with empirical formulas (Gons, 1999; Kirk, 2010).

Measurements of chlorophyll fluorescence have been used to non-invasively assess photosynthesis and physiological changes in photosynthetic organisms for more than 30 years (Conroy et al., 1986; Song et al., 2016; Wang et al., 2018; Zhao et al., 2018; Shang et al., 2019). We previously reported that the fluorescence parameter Fo could be used to determine Chl *a* contents or cell densities of pure cultured microalgae or a diverse phytoplankton community (mainly microalgae) from nature (Chen et al., 2017). Estimated Chl *a* could subsequently be used to model primary productivity. The chlorophyll fluorescence–induced dynamic curve (also known as the OJIP curve) measured after dark adaptation can reflect the original photochemical reaction of PS II and changes to the function and structure of photosystems (Krause and Weis, 1991; Strasserf et al., 1995; Strasser et al., 2004). The analysis of OJIP curves under different environmental conditions can further reveal the effects of environmental factors on photosynthetic systems (especially PS II), and the adaptation mechanism of plants or algae to different environmental factors. In addition, our previous studies show that OJIP curves could evaluate and reflect the photosynthetic electron transport, photosynthetic efficiency, and oxygen releasing ability of photosynthetic algae, which are key factors related to primary productivity (Zhang et al., 2017, 2021). Thus, the OJIP curve may be a potential tool for estimation of water primary productivity.

Algal blooms are marked by exceptional increases in the biomass of photosynthetic algae, especially the harmful algal species in freshwater and coastal systems (Lehahn et al., 2014). Algal blooms pose a severe threat to these systems, causing oxygen depletion, aquatic animal asphyxiation, and generation of foul odors and toxins, which has caused great harm to aquatic ecosystem (Chen et al., 2011, 2014). Thus, it is essential to establish an early warning technology for algal blooms so as to detect the increasing harmful algal biomass and give early warning before ecological harm caused by algal blooms, which can also provide early information for the prevention and control of algal blooms. Our previous study established an early warning method based on the significant correlation between the Fo and cell density of specific algae leading to algal blooms in algal bloom monitoring (Chen et al., 2017).

In the current study, we examine the relationship between the area under the OJIP curve and both Chl *a* content and cell density for pure microalgal cultures as well as mixed cultures and samples from natural ponds. We establish a method for estimating either Chl *a* content or cell density by fitting empirical relationships with the OJIP curve integral. We further modified and validated this method using withheld samples and showed how estimated OJIP curve integrals and Chl *a* content can be used to calculate primary productivity in water samples, which also provides a potential tool for health assessment of natural water and early warning of algal blooms.

## Materials and Methods

### Algal Strains and Culture Conditions

Algal strains used in the experiment are listed in Table 1. The strains Synechocystis PCC 6803, Synechococcus PCC 7942, and *Chlorella* sp. C2 were cultured in BG11 medium (Stanier et al., 1971; Singh et al., 2018) at 30°C and continuous illumination of 30 μmol photons m^–2^ s^–1^. *Chlamydomonas reinhardtii* CC 125 and *C. reinhardtii* CC 4533 were cultured in TAP medium (Tolleter et al., 2011) at 25°C with a continuous illumination of 50 μmol photons m^–2^ s^–1^. Cultures were subjected to continuous shaking at 120 rpm and then harvested at 96 h by centrifuging at 3000 *g* for 3 min at 20°C. The resulting pellets were resuspended with fresh medium to various cell densities.

**TABLE 1:** Algal species used in this study.

Phylum	Algal name	Medium
Cyanophyta	*Synechocystis* sp. PCC 6803	BG11
	*Synechococcus* sp. PCC 7942	BG11
Chlorophyta	*Chlorella* sp. C2	BG11
	*Chlamydomonas reinhardtii* CC125	TAP
	*Chlamydomonas reinhardtii* CC4533	TAP

### Water Sample Collection

Using NisKin water bottles (KC-Denmark, Denmark), water samples from six sampling points (50 mL of each sampling point) at a depth of 1 m were collected in December of 2019 from a natural aquaculture pond in Wuhan, Hubei Province, China. Water samples were centrifuged at 3000 *g* for 5 min at 20°C to generate desired cell densities (10^6^ cell mL^–1^) and subjected to measurements of OJIP, Chl *a*, and cell density. In addition, measurements were made for a mixed algal culture inoculated with Synechocystis PCC 6803, Synechococcus PCC 7942, *Chlorella* sp. C2, *C. reinhardtii* CC 125, and *C. reinhardtii* CC 4533, to investigate the correlation between cell densities and the integral areas of the OJIP curve for mixed communities. Finally, a separate sample of the natural community was centrifuged at 3000 *g* for 5 min at 20°C to generate different cell densities to measure the sensitivity of OJIP curves to varying levels of known Chl *a* content.

### OJIP Curve Measurement

OJIP curves were measured using an AquaPen-C AP-C 100 (Photon Systems Instruments, Brno, Czech Republic). Three milliliters of each sample were dark-adapted for 20 min and subsequently exposed to 620 nm light with a pulse-light intensity of 1800 μmol photons m^–2^ s^–1^ and an actinic light intensity of 50 μmol m^–2^ s^–1^. Origin Pro 8 (OriginLab Corporation, Northampton, Massachusetts, United States) was used to draw the OJIP curve and calculate its integral area.

### Cell Density Measurement

The microfluidic particle analysis system (CytoFlex S, Beckman Coulter, Brea, United States) was used for cell density determination. One milliliter of each sample was filtered through 200 UNIT mesh gauze before measurement. The sample flow rate was set to 10 μL min^–1^ during the measurement, and 10,000 cells were collected per sample. The cell density was calculated by the recorded cell number and sample volume.

### Chl *a* Content Measurement

Chlorophyll *a* concentration was measured as described by Zhang et al. (2013) with some revision. One milliliter of each sample was centrifuged at 10,000 *g* for 3 min at room temperature. Cell pellets were resuspended in 1 mL of 100% (v/v) methanol and stored at 4°C in darkness for 24 h. Pigment extract liquor was subsequently collected by centrifuging samples at 10,000 *g* for 3 min at room temperature, and the optical density (OD) was measured at 652.4 and 665.2 nm using a spectrophotometer. Chl *a* content was calculated using the following formula (Lichtenthaler, 1987):

(1) c(Chla)(mgmL)-1=16.72×OD-665.29.16×OD.652.4

### Primary Productivity Calculation

We estimated the primary productivity of water samples as following formula:

(2) P=K×r×c⁢(C⁢h⁢l⁢a)×D⁢H,

where P represents primary productivity (mg C m^–3^ d^–1^), r represents the assimilation coefficient (mg biomass h^–1^mg^–1^ Chl *a*), c (Chl *a*) represents the content of Chl *a* (mg m^–3^), DH represents sunshine time (h d^–1^), and K represents the experience constant (Tao, 2015; Chen et al., 2017).

The oxygen method (also called the dark and light bottle method) was also used to estimate the water primary productivity according to Wen et al. (2003) and Qi et al. (2015). The basic principle of the oxygen method is to determine the oxygen content in the water to determine the net production of oxygen (mg O_2_ m^–3^ d^–1^) and then use the photosynthesis equation to calculate the primary production (mg C m^–3^ d^–1^), and 1 mg O_2_ = 0.375 mg C/PQ, where the average photosynthetic quotient (PQ) of the natural phytoplankton community is set to 1.25 (Williams et al., 1979). A pond in Henan University, Kaifeng, Henan Province, China, was used for estimating the primary productivity using the oxygen method in November of 2020, and the water samples collected from this pond were also used for measuring Chl *a* contents and OJIP curves as well as estimating the primary productivity using the formula (2).

### Statistical Analysis

Each result shown is the mean of at least three biological replicates and standard deviation. Data were analyzed using the software program SPSS-24 (IBM), and the *t*-test was used to determine the mean and standard deviation of repeated studies. The correlation between integral of OJIP curve and Chl *a* contents or cell density or primary productivity as well as between the primary productivity estimated using the integral area of OJIP curve and that measured using the conventional oxygen method, were assessed by Pearson’s correlation test, and correlation was considered to be significant at *p* < 0.05 or *p* < 0.01, where *p* < 0.05 means significant correlation and *p* < 0.01 means extremely significant correlation.

## Results

### The Integral Area of the OJIP Curve Is Significantly Correlated With Chl *a* Content and Cell Density in Pure Cultures of Photosynthetic Microalgae

**FIGURE 1:**
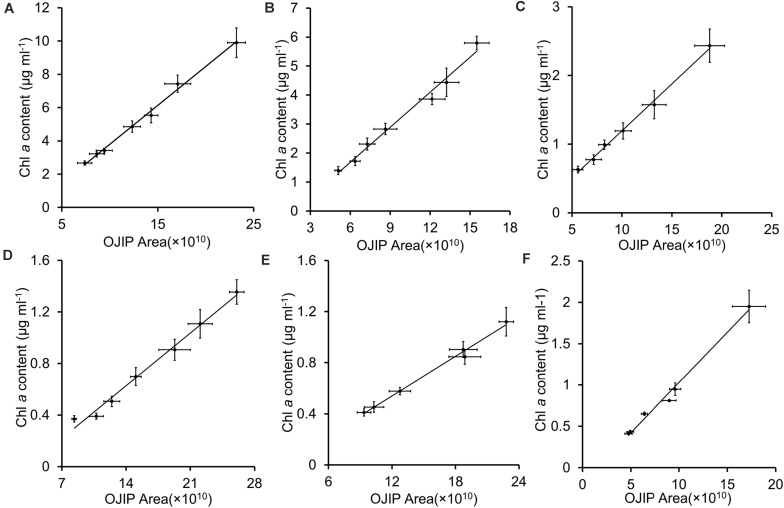
The integral area of the OJIP curve–Chl *a* content linear fits for five representative algal strains and their mixed culture. **(A)** *Chlamydomonas reinhardtii* CC 4533; **(B)** *Chlamydomonas reinhardtii* CC 125; **(C)** *Chlorella* sp. C2; **(D)** *Synechococcus* PCC 7942;**(E)** *Synechocystis* PCC 6803; **(F)** Mixed culture of five algal strains. Each point represents a sample from a different time point in each culture’s growth curve.

**FIGURE 2:**
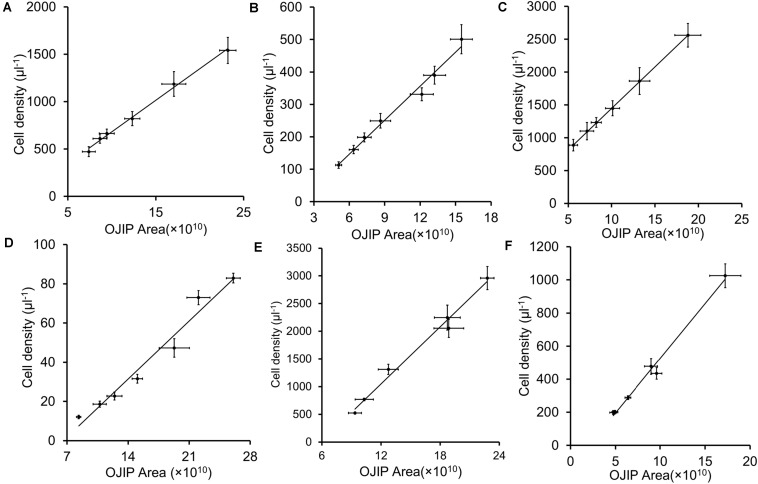
The integral area of OJIP curve–cell density linear fits for five representative algal strains and their mixed culture. **(A)** *Chlamydomonas reinhardtii* CC 4533; **(B)** *Chlamydomonas reinhardtii* CC 125; **(C)** *Chlorella* sp. C2; **(D)** *Synechococcus* PCC 7942; **(E)** *Synechocystis* PCC 6803; **(F)** Mixed culture of five algal strains. Each point represents a sample from a different time point in each culture’s growth curve.

Integrals of OJIP curves were positively correlated with Chl *a* contents for each pure culture of photosynthetic algae, using data from several time points in algal growth (Figures 1A–E and Table 2, *r* > 0.9, *p* < 0.01). Similarly, positive correlations were identified between the integral areas of OJIP curves and cell density in each pure algal culture (Figure 2 and Table 2, *r* > 0.9, *p* < 0.01). Compared with the results measured using traditional methods, estimates of Chl *a* and cell density based on observed correlations deviated from observed values by 1–9% (Table 3).

**TABLE 2:** The integral area of the OJIP curve – Chl *a* content, cell density, or primary productivity correlation equations of pure cultured algae, mixed culture, or natural water (Wuhan).

Algae strain	Integral area (x) – Chl *a* content (y)		Integral area (x) –cell density (y)		Integral area (x) –primary productivity (y)	
			
	Correlation equations	R^2^	Correlation equations	R^2^	Correlation equations	R^2^
PCC 6803	*y* = 0.0517x - 0.0811	0.9919	*y* = 171.52x – 1008.8	0.9865	-	-
PCC 7942	*y* = 0.0583x - 0.1871	0.9882	*y* = 4.2281x – 27.724	0.9687	-	-
C2	*y* = 0.1365x - 0.1706	0.9957	*y* = 126.46x + 185.22	0.9998	-	-
CC125	*y* = 0.4034x - 0.7406	0.9845	*y* = 34.967x – 63.374	0.9854	-	-
CC4533	*y* = 0.4675x - 0.8785	0.9947	*y* = 66.543x + 16.091	0.9954	-	-
Mixed culture	*y* = 0.1215x - 0.1844	0.9901	*y* = 66.133x – 135.27	0.9883	-	-
Natural water (Wuhan)	*y* = 0.594x - 0.9021	0.9967	*y* = 145x + 59.542	0.9977	*y* = 0.1423x - 0.2161	0.9967

**TABLE 3:** The validation of using the integral area of the OJIP curve for determining the Chl *a* content and cell density in algae under pure culture.

Algae strain	Integral area (× 10^10^)	Calculated Chl *a* content by measuring integral area (mg L^–^^1^)	Measured Chl *a* content (mg L^–^^1^)	Deviation	Calculated cell density by measuring integral area (cell μL^–^^*l*^)	Measured cell density (cell μL^–^^*l*^)	Deviation
PCC 6803	17.37 ± 0.33	0.82 ± 0.02	0.79 ± 0.04	3.59%	1969.77 ± 56.775	2048.76 ± 290.98	3.86%
PCC 7942	19.63 ± 0.04	0.96 ± 0.003	0.90 ± 0.05	5.93%	55.27 ± 0.18	51.26 ± 4.32	7.82%
C2	24.49 ± 0.27	3.17 ± 0.04	2.92 ± 0.04	8.54%	3281.86 ± 33.98	3348.50 ± 145.73	1.99%
CC125	13.50 ± 0.16	4.71 ± 0.06	4.30 ± 0.35	9.47%	408.69 ± 5.47	405.35 ± 10.65	0.83%
CC4533	7.63 ± 0.41	2.69 ± 0.19	2.47 ± 0.28	9.09%	539.83 ± 0.42	534.22 ± 44.18	1.05%

### Estimates of Photosynthetic Microalgal Cell Densities in Natural Water

**FIGURE 3:**
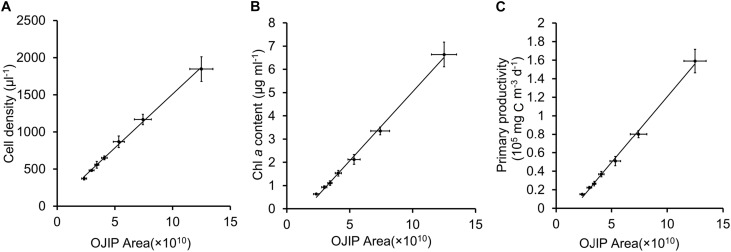
Linear fits for the integral area of OJIP curve and cell density **(A)**, Chl *a* content **(B)** or primary productivity **(C)** for samples of pond water in Wuhan City.

In mixed microalgal cultures, there was a positive linear correlation between integrals of OJIP curves and microalgal cell density (Figure 2F and Table 2, *r* > 0.9, *p* < 0.01), indicating that algal cell growth in complex water can be evaluated by the integral area of OJIP curve. For natural water samples from an aquaculture pond in Wuhan, China, integrals of OJIP curves were also positively correlated with photosynthetic microalgal cell density in natural water samples (Figure 3, *r* > 0.9, *p* < 0.01; Table 2).

The feasibility of using the integral area of the OJIP curve for estimating microalgal cell density in natural water was further assessed using another water sample collected in December 2019 from the same natural aquaculture pond. The predicted cell density of this sample using the fitted relationship deviated from the observed value determined by traditional cell counting by 2.41% (Table 4), indicating that OJIP curve analysis is a reasonable approach for assessing photosynthetic microalgal cell density in natural water.

**TABLE 4:** The validation of using the integral area of the OJIP curve for determining the Chl *a* content, cell density, and primary productivity in the pond of Wuhan City.

Integral area (× 10^10^)	Calculated Chl *a* content by measuring integral area (mg L^–^^1^)	Measured Chl *a* content (mg L^–^^1^)	Deviation	Calculated cell density by measuring integral area (cell μL^–^^*l*^)	Measured cell density (cell μL^–^^*l*^)	Deviation	Estimate primary productivity by measuring integral area (10^5^mg C m^–^^3^ d^–^^1^)	Measured primary productivity (10^5^mg C m^–^^3^ d^–^^1^)	Deviation
12.40 ± 0.13	6.46 ± 0.08	6.56 ± 0.24	1.46%	1857.30 ± 19.12	1813.59 ± 35.93	2.41%	1.55 ± 0.02	1.57 ± 0.06	1.45%
									

### Estimates of Primary Productivity in Natural Water Samples Using the OJIP Curve

Chl *a* content was significantly correlated with the integral area of the OJIP curve in the mixed community cultures (Figure 2 and Table 2, *r* > 0.9, *p* < 0.01), indicating that the integral area of OJIP curve is an efficient index for estimating the Chl *a* content in complex water. A significant positive correlation was also identified between the integral area of the OJIP curve and Chl *a* content in natural water samples (Figure 3, *r* > 0.9, *p* < 0.01). Furthermore, the integral area of the OJIP curve and primary productivity were also correlated (Figure 3, *r* > 0.9, *p* < 0.01; Table 2).

Another water sample was collected from the same natural aquaculture pond in Wuhan, China, to assess the feasibility of estimating the primary productivity using the integral area of the OJIP curve in natural water. There was no significant difference between the predicted and observed primary productivity in the natural water validation sample (1.45% deviation; Table 4). Thus, measuring the integral area of the OJIP curve is a reliable method for assessing the primary productivity of the natural ponds.

**FIGURE 4:**
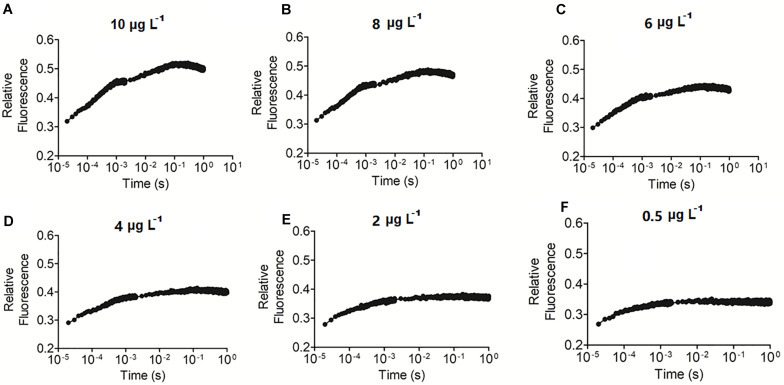
Precision analysis of the OJIP curve method for detecting Chl *a*. **(A–F)** represents the OJIP curve of water samples with 0.5–10 μg L^– 1^ Chl a content, respectively.

OJIP curve integrals were relatively sensitive to changes in Chl *a* contents (Figure 4). Sensitivity analysis revealed that Chl *a* values as low as 0.5 μg L^–1^ could be detected.

### Modification and Validation of the OJIP Curve Method

**FIGURE 5:**
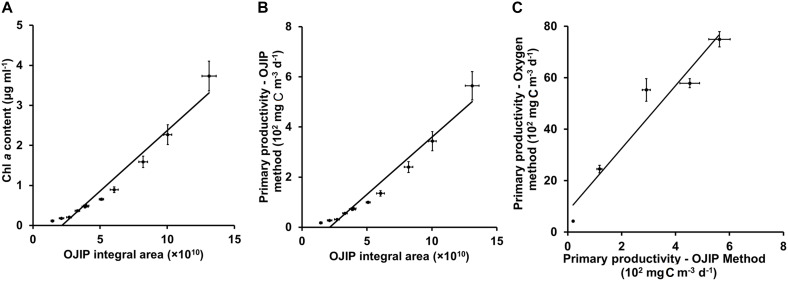
Linear fits for the integral area of the OJIP curve and Chl *a* content **(A)** or calculated primary productivity from Chl *a* content **(B)**, and for the calculated primary productivity by the OJIP curve method and measured primary productivity by the oxygen method **(C)** for samples of pond water in Kaifeng City. The basic principle of the oxygen method is to determine the oxygen content in the water to determine the net production of oxygen (mg O_2_ m^– 3^ d^– 1^). Then, we use the photosynthesis equation to calculate the total primary production (mg C m^– 3^ d^– 1^).

The conventional oxygen method was used to further modify the method based on the OJIP curve in this study. In a pond of Henan University in Kaifeng, China, water primary productivity was measured using the oxygen method. At the same time, the primary productivity of water samples collected from this pond was estimated using the integral area of the OJIP curve (Figures 5A,B, *r* > 0.9, *p* < 0.01; Table 5). Furthermore, the primary productivity estimated using the integral area of the OJIP curve was significantly correlated with the primary productivity measured using the conventional oxygen method (Figure 5C, *r* > 0.9, *p* < 0.01). Accordingly, the calculation formula of primary productivity established by the integral area of the OJIP curve is further modified using the oxygen method (Table 5).

**TABLE 5:** The parameter modification and validation of the integral area of OJIP curve method for estimating primary productivity by the oxygen method in the pond of Kaifeng City.

Integral area (x) –		Integral area (x) –		Primary productivity		Integral area (x) –		Integral area (× 10^10^)	Estimate primary productivity by measuring integral area (10^2^ mg C m^–^^3^ d^–^^1^)	Measured primary productivity by Oxygen method (10^2^ mg C m^–^^3^ d^–^^1^)	Deviation
Chl *a* content (y)		primary		by OJIP method (x) –		modified primary					
		productivity (y)		Primary productivity		productivity (y)					
				by Oxygen method (y)							
			
Correlation equation	R^2^	Correlation equation	R^2^	Correlation equation	R^2^	Correlation equation	R^2^				
*y* = 0.3025x – 0.65	0.9577	*y* = 0.4576x – 0.9835	0.9577	*y* = 12.199x + 8.1251	0.935	*y* = 5.5822x – 3.8725	0.935	6.38 ± 0.7	31.74 ± 3.48	31.05 ± 5.94	2.17%

Water sample was collected from the same pond to verify the modified calculation formula of primary productivity, and there was no significant difference between the results from modified calculation formula and the oxygen method (2.17% deviation; Table 5), proving the practicality of the modified OJIP curve method for assessing the primary productivity of the natural ponds.

## Discussion

Algae generate most of the primary productivity in aquatic ecosystems, particularly in deep areas where photosynthetic microalgae are almost the only autotrophs (Redfield et al., 1963). Common methods for measuring algal cell density are either prohibitively time-consuming (e.g., involve direct counting with a cell counter and microscope) or inaccurate (e.g., involve indirect estimates through spectrophotometry) (Lund et al., 1958) and unable distinguish between algal and non-algal cells or other impurities.

By contrast, Chl *a* content is strongly linked to the species and quantity of photosynthetic algae in aquatic ecosystems and a common indicator of phytoplankton biomass (Lorenzen, 1966; Paerl et al., 1976), which is significantly positively correlated with water primary productivity (Platt and Sathyendranath, 1993). However, there is some criticism of the primary methods used to measure Chl *a* content, including the colorimetry and fluorescence method (Kruskopf and Flynn, 2006). For example, the colorimetry method is technically intricate and the organic solvent for extracting the chlorophyll is unsuitable for field applications. Furthermore, in the fluorescence method, the detection error of Chl *a* content in natural communities is usually large compared with pure cultures. Proposed remote sensing methods using reflectance to estimate Chl *a* concentrations (Tang et al., 2012) require large initial investments in capital and technical expertise, limiting the popularity of this technology.

In the present study, both cell density and Chl *a* content were linearly associated with the integral area of the OJIP curve in both pure algal and mixed cultures or natural communities (Figures 1–3 and Table 2). The OJIP curve, a classic fluorescence-kinetics curve, integrates many factors related to the activity of photosynthesis (especially PS II), is obtained in a relatively short amount of time and is non-destructive (Maxwell and Johnson, 2000). Based on the strong predictive relationships observed in this study (Table 2), Chl *a* contents or cell densities of microalgae in pure culture and a natural pond can be readily determined using the area under the OJIP curve (Tables 3, 4).

The classic gold standard method for estimating primary productivity is the carbon isotope tracer method (Peterson, 1980; López-Sandoval et al., 2018). Due to a series of shortcomings in the isotope tracer method (e.g., it is a costly, time-consuming, and complex operation), some methods, including the oxygen, Chl *a*, and remote sensing methods, were developed (Bott et al., 1978; Gons, 1999; Joint et al., 2002; Vase et al., 2018). Chl *a* content is a basic parameter for estimating primary productivity in aquatic systems and has been verified by the carbon isotope method (Pei et al., 2018). For example, Yang et al. (2002) estimate the primary productivity of Tai Lake based on Chl *a* content. We previously developed an empirical equation between chlorophyll fluorescence parameter Fo and Chl *a* content using natural water samples and used this method to estimate primary productivity (Chen et al., 2017). In the present study, we established that the integral area of the OJIP curve is also associated with Chl *a* content and estimated primary productivity. Both chlorophyll fluorescence methods are the extension of the Chl *a* method, and the significant linear correlation (*r* > 0.9) between Chl *a* contents and fluorescence parameters guarantee the reliability of chlorophyll fluorescence methods. However, Fo is known to increase significantly in stressed organisms (Hong and Xu, 1999; Kuckenberg et al., 2009; Zhang et al., 2017), making the Fo method suitable only for healthy, unstressed microalgae. Unlike Fo, the OJIP curve integral area decreases correspondingly with a decrease of Chl *a* content and cell density due to cell stress, suggesting that the OJIP curve-based method could also be used to assess stressed microalgae. The Chl *a* content in most lakes ranges from 3 to 994 μg L^–1^, and traditional methods tend to have a low detection sensitivity (10 μg L^–1^ Chl *a*), requiring that samples be concentrated before measurements are taken. The OJIP curve method was found to have a relatively high detection sensitivity (0.5 μg L^–1^ Chl *a*) (Figure 4). Thus, samples may not need to be concentrated before using this method.

Based on Chl *a* content, water primary productivity can be rapidly and simply measured according to some empirical formulas (Esaias et al., 1998; Carder et al., 2004). Several empirical formulas and models have been reported (Arst et al., 2008; Kauera et al., 2013). However, a single empirical formula is not always appropriate, given the variability in environmental conditions in natural systems, in which the relationship between Chl *a* and productivity is unlikely to be constant. Platt and Sathyendranath (1993) established that primary productivity could be attributed to the product form of some factors, including average production of water column, surface light intensity, and chlorophyll content based on an analysis of a large number of primary productivity models. For different microalgal communities with different environmental factors, the appropriate adjustments of empirical formula should be made to be optimal. In this study, the oxygen method was further used to modify the empirical formula established by the OJIP curve method, and the practicability of the modified empirical formula was proved (Figure 5 and Table 5).

Recently, due to the ecological and economic impacts of algal blooms, there is increased interest in developing early warning technologies (Campbell et al., 2013; Chen et al., 2015). Chen et al. (2017) establish that the cell density fluctuation of harmful algal species in aquatic systems could be timely detected by monitoring Fo. The present study provides another chlorophyll fluorescence–based indicator that can be used to monitor algal blooms. Based on the strong relationship between the OJIP curve integrals and cell densities of algae common in algal blooms, the method could be used to anticipate an algal bloom outbreak with consistent monitoring.

## Conclusion

**FIGURE 6:**
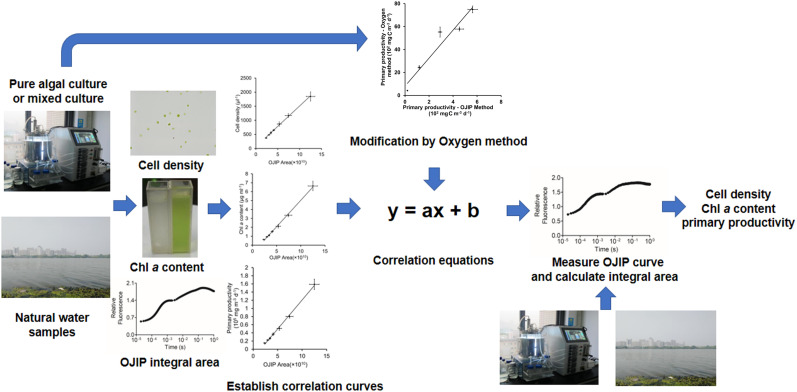
Flow diagram for recommended application of the OJIP curve method for determining the cell density, Chl *a* content, and water primary productivity.

In conclusion, we present a rapid and non-invasive technique to estimate primary productivity in aquatic ecosystems based on OJIP curves (Figure 6). First, linear equations are established based on the relationship between the OJIP curve integrals and Chl *a* contents or cell densities. Based on these initial intensive samples, the Chl *a* content or cell density may be efficiently measured using this established relationship. This OJIP curves method can be further modified and used to estimate primary productivity in aquatic ecosystems based on the relevant empirical formula. We expect this method to be potentially effective in health assessment of natural water and early detection efforts for algal blooms.

## Data Availability Statement

The original contributions presented in the study are included in the article/supplementary material, further inquiries can be directed to the corresponding author.

## Author Contributions

QW conceived and designed the experiments and provided reagents and materials. HC, KL, and CX performed the experiments. HC and KL analyzed the data. HC wrote the manuscript and QW revised it. All of the authors read and approved of the final version to be published.

## Conflict of Interest

The authors declare that the research was conducted in the absence of any commercial or financial relationships that could be construed as a potential conflict of interest.
